# Supplementary material for: A Likelihood Approach for Real-Time Calibration of Stochastic Compartmental Epidemic Models
Source: PLoS Comput Biol. 2017 Jan 17;13(1):e1005257. doi: 10.1371/journal.pcbi.1005257 (PMC5240920; doi:10.1371/journal.pcbi.1005257)
Supplement: S1 File — (TAR.GZ) [file pcbi.1005257.s014.tar.gz › HSPH_Online-SI-Revision/IPoi09/n1-severe/n1-severe_BENCH_table0.pdf]

| | | | | | | | | | | | | | | | | | | | | | | | | | | | |
|---|---|---|---|---|---|---|---|---|---|---|---|---|---|---|---|---|---|---|---|---|---|---|---|---|---|---|---|
| 1 | R0 | {1.51081, 13.7078} | {2.04072, 15.6294} | {{1.86094, 12.8385}, {2.26161, 19.3849}} | {35.0745, 16.4164} | inf | 54. | 131.911 | {74., 185.} | 145.403 | 0.369049 | newly | 34. | 53.625 | {32., 75.} | 63.0777 | 0.203531 | Reff | 1.05303 | 0.780635 | {0.536002, 1.15375} | 30.1819 | ar | 656. | 802.518 | {738., 863.} | 22.335 |
| 2 | R0 | {1.73182, 11.3873} | {2.41911, 19.2001} | {{2.37462, 18.743}, {2.45321, 19.5287}} | {39.6862, 68.61} | inf | 80. | 173.085 | {123., 224.} | 116.743 | 0.322149 | newly | 53. | 61.0845 | {40., 84.} | 27.9564 | 0.111807 | Reff | 1.15166 | 0.763072 | {0.448868, 1.22979} | 40.17 | ar | 653. | 880.817 | {860., 904.} | 34.8877 |
| 3 | R0 | {1.50449, 10.3474} | {2.09268, 16.2595} | {{1.8484, 12.6353}, {2.26161, 19.3849}} | {39.0953, 57.1359} | inf | 60. | 148.787 | {108., 204.} | 148.033 | 0.37633 | newly | 43. | 63.5902 | {48., 80.} | 51.3915 | 0.175383 | Reff | 1.08474 | 1.03819 | {0.667536, 1.45422} | 23.5579 | ar | 651. | 820.197 | {768., 870.} | 25.9903 |
| 4 | R0 | {1.63493, 9.1955} | {2.80039, 19.7371} | {{2.7811, 19.6712}, {2.85956, 19.984}} | {71.2851, 114.639} | inf | 87. | 239.423 | {171., 296.} | 181.868 | 0.461066 | newly | 59. | 85.1268 | {68., 105.} | 51.9694 | 0.193897 | Reff | 1.05126 | 1.23054 | {0.65634, 1.87569} | 37.5132 | ar | 663. | 924.746 | {905., 941.} | 39.4791 |
| 5 | R0 | {1.53822, 8.05553} | {2.67531, 18.8118} | {{2.42074, 16.1449}, {2.79269, 19.984}} | {73.9227, 133.527} | inf | 55. | 234.716 | {192., 280.} | 326.757 | 0.622626 | newly | 40. | 82.7612 | {66., 100.} | 107.425 | 0.311711 | Reff | 1.01061 | 0.973446 | {0.522847, 1.53517} | 28.8296 | ar | 621. | 911.731 | {879., 934.} | 46.8166 |
| 6 | R0 | {1.69374, 17.0793} | {2.05366, 19.3422} | {{1.98971, 19.0665}, {2.11476, 19.6444}} | {21.25, 13.2492} | inf | 97. | 128.691 | {61., 180.} | 51.2277 | 0.199267 | newly | 40. | 49.2727 | {26., 70.} | 41.7273 | 0.167989 | Reff | 1.25676 | 1.26955 | {0.824756, 1.72353} | 23.7925 | ar | 662. | 808.145 | {759., 851.} | 22.0764 |
| 7 | R0 | {1.42179, 11.8031} | {2.24009, 17.9551} | {{2.14288, 16.1983}, {2.30371, 19.6625}} | {57.5539, 52.1217} | inf | 57. | 137.068 | {75., 195.} | 143.205 | 0.368368 | newly | 30. | 50.4068 | {25., 72.} | 75.8192 | 0.242656 | Reff | 0.963974 | 0.67503 | {0.412239, 0.940351} | 36.8082 | ar | 517. | 850.797 | {813., 893.} | 64.5641 |
| 8 | R0 | {1.46683, 6.95875} | {2.89605, 18.8489} | {{2.8568, 18.2812}, {2.93978, 19.9817}} | {97.4357, 170.866} | inf | 92. | 250.192 | {189., 297.} | 171.948 | 0.425471 | newly | 97. | 90.6164 | {72., 106.} | 12.4841 | 0.0601511 | Reff | 0.815557 | 1.06177 | {0.531365, 1.69625} | 47.7316 | ar | 653. | 929.562 | {911., 944.} | 42.3525 |
| 9 | R0 | {1.34525, 11.505} | {2.10414, 18.5026} | {{1.97176, 16.2766}, {2.1968, 19.9579}} | {56.4123, 60.8227} | inf | 47. | 146.574 | {82., 198.} | 215.208 | 0.486943 | newly | 31. | 55.5902 | {38., 73.} | 85.1401 | 0.272057 | Reff | 1.01701 | 1.05505 | {0.691887, 1.52} | 23.2733 | ar | 532. | 819.885 | {770., 859.} | 54.1138 |
| 10 | R0 | {1.77958, 14.372} | {2.42778, 19.3773} | {{2.41419, 19.1373}, {2.45321, 19.4475}} | {36.4242, 34.8269} | inf | 110. | 177.368 | {120., 229.} | 66.8048 | 0.227957 | newly | 47. | 62.0294 | {38., 84.} | 42.1777 | 0.163566 | Reff | 0.948516 | 0.905612 | {0.503358, 1.59812} | 36.8737 | ar | 671. | 883.529 | {864., 901.} | 31.6735 |
| 11 | R0 | {1.60334, 19.9548} | {2.00256, 19.2058} | {{1.98971, 18.8819}, {2.036, 19.6444}} | {24.8993, 3.75372} | inf | 94. | 117.067 | {72., 155.} | 34.6099 | 0.132481 | newly | 31. | 40.7167 | {26., 55.} | 41.0215 | 0.147254 | Reff | 1.08867 | 0.951954 | {0.573988, 1.55993} | 32.8353 | ar | 617. | 797.45 | {754., 837.} | 29.2464 |
| 12 | R0 | {1.68623, 7.58585} | {2.82981, 15.4493} | {{2.7499, 15.0558}, {2.96947, 15.963}} | {67.8185, 103.659} | inf | 74. | 235.352 | {180., 285.} | 218.805 | 0.49413 | newly | 59. | 99.7042 | {76., 122.} | 69.6586 | 0.222954 | Reff | 0.881898 | 0.842495 | {0.47689, 1.38423} | 36.1367 | ar | 640. | 926.507 | {912., 944.} | 44.7667 |
| 13 | R0 | {1.82689, 18.0022} | {2.27607, 19.2152} | {{2.25962, 18.788}, {2.30371, 19.6625}} | {24.5869, 6.9697} | inf | 136. | 164.597 | {116., 209.} | 32.3174 | 0.161996 | newly | 49. | 58.6774 | {37., 78.} | 33.2456 | 0.171073 | Reff | 1.10344 | 0.969396 | {0.533613, 1.47909} | 32.2561 | ar | 673. | 842.21 | {821., 884.} | 28.239 |
| 14 | R0 | {1.75911, 9.16883} | {2.7864, 18.1221} | {{2.57305, 15.7308}, {2.93978, 19.9817}} | {58.398, 97.6487} | inf | 93. | 236.703 | {140., 300.} | 160.198 | 0.435783 | newly | 75. | 94.6875 | {62., 118.} | 37.3333 | 0.187165 | Reff | 1.16629 | 1.28924 | {0.779025, 1.95732} | 32.5272 | ar | 661. | 920.141 | {889., 945.} | 39.2043 |
| 15 | R0 | {1.46168, 11.3859} | {2.40923, 19.2982} | {{2.37462, 18.743}, {2.42625, 19.5287}} | {64.8263, 69.4917} | inf | 70. | 135.125 | {75., 202.} | 94.3304 | 0.264668 | newly | 36. | 45.2969 | {23., 71.} | 42.5781 | 0.155777 | Reff | 0.809771 | 0.559468 | {0.37519, 0.801699} | 42.6697 | ar | 556. | 882.656 | {865., 901.} | 58.7511 |
| 16 | R0 | {1.53577, 11.7581} | {2.56397, 16.4684} | {{2.19696, 13.3585}, {2.86095, 19.6712}} | {66.9504, 40.6479} | inf | 94. | 206.889 | {115., 268.} | 122.02 | 0.330255 | newly | 55. | 87.1746 | {56., 108.} | 61.7316 | 0.203283 | Reff | 1.05507 | 1.10016 | {0.546799, 2.07162} | 37.3666 | ar | 625. | 895.413 | {847., 938.} | 43.266 |
| 17 | R0 | {1.70698, 9.72924} | {2.81299, 15.8647} | {{2.688, 14.8279}, {2.8939, 16.8013}} | {64.7932, 63.0624} | inf | 101. | 240.653 | {185., 289.} | 138.655 | 0.370921 | newly | 63. | 99.2778 | {80., 122.} | 58.157 | 0.192503 | Reff | 0.913234 | 0.851001 | {0.452066, 1.52215} | 36.5713 | ar | 675. | 924.542 | {905., 944.} | 36.9691 |
| 18 | R0 | {1.5973, 14.931} | {2.02547, 12.7632} | {{1.81647, 10.0503}, {2.24606, 16.4137}} | {26.8061, 19.4399} | inf | 53. | 128.933 | {59., 186.} | 154.34 | 0.42267 | newly | 23. | 71.9667 | {37., 101.} | 222.609 | 0.517452 | Reff | 1.28583 | 1.31317 | {0.826561, 1.85695} | 23.8426 | ar | 632. | 804.117 | {703., 881.} | 27.2336 |
| 19 | R0 | {1.87468, 17.004} | {2.27856, 19.1547} | {{2.26161, 19.0355}, {2.29738, 19.3849}} | {21.544, 12.6481} | inf | 112. | 142.045 | {91., 192.} | 37.3241 | 0.15937 | newly | 45. | 48.8636 | {28., 69.} | 30.4714 | 0.146055 | Reff | 0.987956 | 0.717793 | {0.42961, 1.24985} | 40.7151 | ar | 692. | 845.03 | {824., 891.} | 25.0263 |
| 20 | R0 | {1.84715, 12.531} | {2.3099, 19.1161} | {{2.29738, 19.0355}, {2.37462, 19.4475}} | {25.0519, 52.5502} | inf | 90. | 151.373 | {88., 209.} | 76.9486 | 0.276624 | newly | 55. | 52.7164 | {33., 76.} | 27.5441 | 0.167975 | Reff | 0.936505 | 0.790271 | {0.450059, 1.27734} | 38.6037 | ar | 661. | 850.224 | {832., 897.} | 31.4901 |
| 21 | R0 | {1.75938, 13.5959} | {1.95345, 9.48543} | {{1.82266, 8.17412}, {2.02235, 10.6804}} | {11.1464, 30.7884} | inf | 92. | 84.1091 | {35., 138.} | 37.747 | 0.220114 | newly | 50. | 53. | {21., 88.} | 43.4545 | 0.214125 | Reff | 1.1911 | 0.669144 | {0.427456, 0.969655} | 46.2306 | ar | 620. | 784.691 | {731., 831.} | 26.563 |
| 22 | R0 | {1.63456, 10.7469} | {2.41029, 19.424} | {{2.37462, 19.3966}, {2.42205, 19.4475}} | {47.4582, 80.7409} | inf | 85. | 167.03 | {129., 212.} | 100.088 | 0.296196 | newly | 61. | 58.1045 | {39., 79.} | 19.7211 | 0.0982667 | Reff | 0.81728 | 0.870576 | {0.4723, 1.58886} | 42.1616 | ar | 654. | 878.045 | {852., 901.} | 34.2576 |
| 23 | R0 | {1.62702, 19.1392} | {1.96287, 12.4755} | {{1.83592, 11.1369}, {2.12134, 14.3165}} | {20.6421, 34.8171} | inf | 97. | 98.3269 | {51., 149.} | 31.1856 | 0.149468 | newly | 37. | 49.7308 | {25., 71.} | 50.8316 | 0.186664 | Reff | 1.02014 | 0.718595 | {0.527659, 0.977749} | 33.1474 | ar | 600. | 779.942 | {728., 837.} | 29.9904 |
| 24 | R0 | {1.70466, 12.1202} | {2.31617, 18.2237} | {{2.22832, 17.1868}, {2.45321, 19.4475}} | {35.8729, 50.3581} | inf | 70. | 102.462 | {59., 155.} | 53.0549 | 0.170014 | newly | 32. | 36.0923 | {20., 55.} | 35.1923 | 0.142995 | Reff | 0.946086 | 0.573496 | {0.350582, 0.746632} | 49.9604 | ar | 613. | 862.231 | {833., 891.} | 40.6575 |
| 25 | R0 | {1.61735, 11.3169} | {2.18583, 19.8558} | {{2.12588, 19.2116}, {2.1968, 19.9579}} | {35.1488, 75.4529} | inf | 103. | 134.786 | {81., 191.} | 40.0485 | 0.157785 | newly | 62. | 44.6964 | {27., 63.} | 30.0979 | 0.194093 | Reff | 0.826466 | 0.806854 | {0.450344, 1.33346} | 36.1624 | ar | 681. | 838.911 | {800., 873.} | 23.1881 |
| 26 | R0 | {1.56977, 12.3035} | {2.32125, 15.0093} | {{2.11132, 12.644}, {2.66225, 19.6025}} | {47.872, 23.4744} | inf | 78. | 145.651 | {78., 223.} | 92.9589 | 0.268838 | newly | 42. | 58.3492 | {30., 83.} | 50.189 | 0.174659 | Reff | 1.03605 | 0.66338 | {0.410665, 0.994862} | 45.7156 | ar | 594. | 865.952 | {817., 912.} | 45.7832 |
| 27 | R0 | {1.46317, 5.8298} | {2.61338, 19.1548} | {{2.46976, 17.5057}, {2.66225, 19.9254}} | {78.6109, 228.567} | inf | 64. | 201.641 | {134., 262.} | 219.165 | 0.494148 | newly | 64. | 71.2813 | {50., 95.} | 25.3906 | 0.111322 | Reff | 0.847175 | 1.01165 | {0.513814, 1.60534} | 46.9142 | ar | 586. | 901.141 | {866., 920.} | 53.7783 |
| 28 | R0 | {1.70002, 14.9806} | {2.648, 17.7816} | {{2.33995, 14.464}, {2.86095, 19.984}} | {55.7626, 21.2676} | inf | 110. | 219.594 | {134., 289.} | 103.347 | 0.299724 | newly | 42. | 87.2464 | {67., 108.} | 110.283 | 0.317144 | Reff | 1.16281 | 1.22089 | {0.648108, 1.94677} | 32.415 | ar | 652. | 907.841 | {876., 941.} | 39.2394 |
| 29 | R0 | {1.52729, 16.1014} | {2.26765, 19.3244} | {{2.25962, 19.0355}, {2.30371, 19.6625}} | {48.4751, 20.0168} | inf | 86. | 117.492 | {64., 185.} | 48.8766 | 0.169571 | newly | 44. | 40.2373 | {18., 66.} | 31.8182 | 0.164772 | Reff | 0.925538 | 0.548561 | {0.363659, 0.776514} | 41.9774 | ar | 592. | 856.068 | {822., 891.} | 44.606 |
| 30 | R0 | {1.72795, 13.5635} | {2.46288, 19.2463} | {{2.41419, 18.743}, {2.52006, 19.5955}} | {42.5321, 41.8979} | inf | 92. | 151.957 | {104., 205.} | 68.4627 | 0.220573 | newly | 43. | 51.3714 | {32., 70.} | 31.3621 | 0.121798 | Reff | 0.865703 | 0.655657 | {0.367929, 1.02603} | 40.8699 | ar | 668. | 884.171 | {859., 910.} | 32.361 |
| 31 | R0 | {1.51591, 18.1544} | {1.89366, 14.0017} | {{1.78373, 11.799}, {2.01238, 16.748}} | {24.919, 22.9909} | inf | 59. | 87.0179 | {28., 164.} | 68.7349 | 0.229565 | newly | 16. | 39.5 | {15., 71.} | 152.232 | 0.363754 | Reff | 1.1051 | 0.710077 | {0.441043, 1.12587} | 40.9023 | ar | 541. | 772.607 | {716., 832.} | 42.8109 |
| 32 | R0 | {1.4282, 10.3534} | {2.20787, 18.528} | {{2.0818, 16.6365}, {2.27287, 19.5128}} | {54.5913, 78.9554} | inf | 38. | 144.617 | {59., 206.} | 287.939 | 0.580154 | newly | 21. | 55.5167 | {20., 74.} | 173.413 | 0.453433 | Reff | 1.1397 | 1.36046 | {0.867513, 1.86764} | 30.7736 | ar | 521. | 841.117 | {792., 874.} | 61.4427 |
| 33 | R0 | {1.56693, 11.3972} | {2.72359, 16.4186} | {{2.46794, 14.1724}, {2.96978, 18.8445}} | {73.8167, 44.1766} | inf | 107. | 223.382 | {168., 285.} | 115.063 | 0.346596 | newly | 62. | 92.8971 | {70., 118.} | 56.8074 | 0.208156 | Reff | 0.965229 | 1.13854 | {0.588474, 1.89006} | 41.4974 | ar | 695. | 914.809 | {880., 947.} | 31.6272 |
| 34 | R0 | {1.50675, 19.8823} | {1.95776, 13.2327} | {{1.8484, 12.3777}, {2.01902, 14.4607}} | {29.9328, 33.4448} | inf | 68. | 56. | {14., 106.} | 46.3609 | 0.284697 | newly | 22. | 25.2542 | {7., 52.} | 59.168 | 0.261807 | Reff | 0.90405 | 0.528515 | {0.381222, 0.697183} | 42.8987 | ar | 587. | 789.525 | {748., 837.} | 34.5018 |
| 35 | R0 | {1.72889, 7.76068} | {2.89529, 17.5993} | {{2.73108, 15.6124}, {2.99088, 18.9406}} | {67.4655, 126.775} | inf | 77. | 250.435 | {135., 311.} | 226.482 | 0.495379 | newly | 62. | 97.4783 | {70., 121.} | 62.7863 | 0.211309 | Reff | 1.04771 | 1.0881 | {0.571654, 2.05384} | 38.0773 | ar | 650. | 930.986 | {908., 950.} | 43.2285 |
| 36 | R0 | {1.62644, 4.12303} | {2.79072, 11.1318} | {{2.5684, 9.75224}, {2.96796, 12.4643}} | {71.5843, 169.99} | inf | 43. | 160.043 | {82., 240.} | 272.193 | 0.540778 | newly | 57. | 85.4 | {44., 127.} | 59.3484 | 0.192828 | Reff | 0.769306 | 0.440572 | {0.300014, 0.642503} | 43.9927 | ar | 652. | 920.557 | {895., 943.} | 41.1897 |
| 37 | R0 | {1.70878, 15.8072} | {2.21768, 19.5839} | {{2.12588, 19.2116}, {2.26161, 19.9579}} | {29.7814, 23.8923} | inf | 84. | 122.317 | {75., 171.} | 51.6629 | 0.1728 | newly | 36. | 40.2698 | {23., 61.} | 32.8483 | 0.135475 | Reff | 0.915906 | 0.606989 | {0.400209, 0.847965} | 37.676 | ar | 674. | 847.27 | {811., 878.} | 25.7077 |
| 38 | R0 | {1.58967, 6.80568} | {2.95259, 18.6054} | {{2.86501, 18.1089}, {2.99088, 18.9406}} | {85.736, 173.381} | inf | 73. | 259.736 | {211., 304.} | 255.803 | 0.545957 | newly | 63. | 92.4444 | {73., 113.} | 47.134 | 0.162429 | Reff | 0.86637 | 0.832394 | {0.457841, 1.45995} | 35.4096 | ar | 610. | 935.806 | {919., 950.} | 53.4107 |
| 39 | R0 | {1.344, 8.19498} | {2.27271, 18.8658} | {{2.21213, 17.5123}, {2.30371, 19.6625}} | {69.1002, 130.212} | inf | 39. | 167.868 | {122., 214.} | 333.522 | 0.642115 | newly | 36. | 61.6176 | {47., 79.} | 75.5719 | 0.270939 | Reff | 0.928704 | 0.99996 | {0.583535, 1.48909} | 28.1674 | ar | 551. | 842.485 | {820., 884.} | 55.6982 |
| 40 | R0 | {1.61509, 7.90825} | {2.0494, 7.61994} | {{1.80312, 5.98216}, {2.13505, 8.31782}} | {26.8909, 10.9061} | inf | 48. | 102.69 | {30., 169.} | 126.509 | 0.338043 | newly | 41. | 78.6379 | {28., 131.} | 104.079 | 0.289297 | Reff | 1.04173 | 0.672665 | {0.418987, 1.07393} | 42.839 | ar | 581. | 815.707 | {752., 861.} | 40.3971 |
| 41 | R0 | {1.60017, 5.90605} | {2.89635, 19.3134} | {{2.81891, 18.4249}, {2.93978, 19.9817}} | {81.0023, 227.011} | inf | 76. | 245.133 | {188., 303.} | 222.544 | 0.499282 | newly | 76. | 83.72 | {64., 105.} | 18.7895 | 0.0773975 | Reff | 0.832088 | 0.889653 | {0.487477, 1.58748} | 41.8039 | ar | 661. | 928.627 | {914., 946.} | 40.4881 |
| 42 | R0 | {1.64436, 15.1337} | {2.02848, 12.0136} | {{1.87584, 10.2217}, {2.2143, 14.4309}} | {23.3599, 20.9021} | inf | 74. | 122.019 | {66., 177.} | 74.1424 | 0.235198 | newly | 39. | 63.8269 | {42., 88.} | 69.4773 | 0.225053 | Reff | 1.11323 | 0.733658 | {0.500836, 1.05924} | 36.6208 | ar | 640. | 805.135 | {759., 861.} | 25.8023 |
| 43 | R0 | {1.48917, 11.917} | {2.12821, 19.5656} | {{2.11476, 19.2116}, {2.1968, 19.9579}} | {42.9123, 64.1821} | inf | 72. | 133.565 | {85., 179.} | 88.3737 | 0.26583 | newly | 36. | 45.5484 | {29., 66.} | 37.6344 | 0.138894 | Reff | 0.927753 | 0.793354 | {0.471591, 1.17581} | 32.2033 | ar | 551. | 826.387 | {792., 866.} | 49.9795 |
| 44 | R0 | {1.65577, 7.89317} | {2.78863, 19.4846} | {{2.73737, 17.9172}, {2.85956, 19.8999}} | {68.4188, 146.854} | inf | 65. | 237.24 | {175., 293.} | 267.282 | 0.561558 | newly | 60. | 83.7867 | {62., 104.} | 44.5778 | 0.172221 | Reff | 1.09446 | 1.04658 | {0.522847, 1.9273} | 36.3425 | ar | 576. | 922.08 | {903., 941.} | 60.0833 |
| 45 | R0 | {1.6909, 12.3275} | {2.55722, 18.8521} | {{2.36153, 17.2664}, {2.66225, 19.9254}} | {51.234, 52.9272} | inf | 82. | 203.833 | {145., 246.} | 150.644 | 0.39627 | newly | 47. | 73.375 | {55., 93.} | 58.5993 | 0.196961 | Reff | 0.968886 | 0.84934 | {0.479205, 1.47785} | 35.915 | ar | 675. | 897.458 | {863., 925.} | 32.9568 |
| 46 | R0 | {1.62351, 16.0698} | {1.97861, 17.2204} | {{1.83921, 14.6061}, {2.11476, 19.5406}} | {21.8724, 11.042} | inf | 58. | 128.426 | {80., 178.} | 124.553 | 0.335845 | newly | 28. | 50.6852 | {29., 69.} | 83.9286 | 0.251632 | Reff | 1.25173 | 0.936391 | {0.61751, 1.38432} | 31.613 | ar | 591. | 793.704 | {738., 839.} | 34.2984 |
| 47 | R0 | {1.52372, 4.38599} | {2.89149, 15.2362} | {{2.84915, 14.6465}, {2.96947, 15.963}} | {89.7652, 247.383} | inf | 71. | 250.471 | {200., 304.} | 252.775 | 0.539155 | newly | 95. | 112.221 | {87., 134.} | 21.8421 | 0.0844319 | Reff | 0.888329 | 1.01475 | {0.544188, 1.79212} | 38.9141 | ar | 618. | 931.015 | {911., 947.} | 50.6496 |
| 48 | R0 | {1.44306, 11.788} | {2.35756, 17.6893} | {{2.20881, 15.6411}, {2.49092, 19.3027}} | {63.3723, 50.0619} | inf | 70. | 146.094 | {88., 212.} | 111.429 | 0.30822 | newly | 36. | 54.1406 | {31., 77.} | 57.7691 | 0.19236 | Reff | 0.883153 | 0.67938 | {0.412826, 1.13939} | 41.8828 | ar | 534. | 869.281 | {824., 905.} | 62.7868 |
| 49 | R0 | {1.63624, 11.3893} | {2.89195, 18.5169} | {{2.75274, 17.3228}, {2.97157, 19.3547}} | {76.7435, 62.5816} | inf | 91. | 238.3 | {178., 293.} | 161.868 | 0.410894 | newly | 55. | 84.8714 | {60., 109.} | 55.7143 | 0.183336 | Reff | 0.927748 | 0.766842 | {0.406612, 1.37287} | 40.1028 | ar | 643. | 933.314 | {919., 946.} | 45.15 |
| 50 | R0 | {1.46113, 11.1322} | {2.25522, 18.6423} | {{2.19504, 17.1709}, {2.30371, 19.6625}} | {54.3478, 67.4632} | inf | 63. | 165.687 | {115., 214.} | 168.633 | 0.452893 | newly | 52. | 62.8209 | {43., 82.} | 31.8886 | 0.168003 | Reff | 1.09731 | 1.09697 | {0.636562, 1.59417} | 29.2259 | ar | 501. | 836.448 | {813., 877.} | 69.7798 |
